# Isolation and Characterization of Microsatellite Loci for *Cotesia plutellae* (Hymenoptera: Braconidae)

**DOI:** 10.3390/insects8020063

**Published:** 2017-06-20

**Authors:** Tiansheng Liu, Fushi Ke, Shijun You, Wenbin Chen, Weiyi He, Minsheng You

**Affiliations:** 1 Institute of Applied Ecology and Research Centre for Biodiversity and Eco-Safety, Fujian Agriculture and Forestry University, Fuzhou 350002, China; liutsfafu@163.com (T.L.); fjkfs@163.com (F.K.); sjyou9@hotmail.com (S.Y.); chenwb63@126.com (W.C.); wy_he@126.com (W.H.); 2 Fujian-Taiwan Joint Centre for Ecological Control of Crop Pests, Fujian Agriculture and Forestry University, Fuzhou 350002, China; 3 Key Laboratory of Integrated Pest Management of Fujian and Taiwan, China Ministry of Agriculture, Fuzhou 350002, China; 4 Department of Botany, University of British Columbia, Vancouver, BC V6T 1Z4, Canada

**Keywords:** microsatellite loci, Hymenoptera, genetic variation, polymorphism

## Abstract

Fourteen polymorphic microsatellite loci were isolated in this transcriptome-based data analysis for *Cotesia plutellae*, which is an important larval parasitoid of the worldwide pest *Plutella xylostella*. A subsequent test was performed for a wild *C. plutellae* population (*N* = 32) from Fuzhou, Fujian, southeastern China, to verify the effectiveness of the 14 microsatellite loci in future studies on *C. plutellae* genetic diversity. The observed number of alleles ranged from two to six. The expected and observed heterozygosity ranged from 0.123 to 0.316 and from 0.141 to 0.281, respectively. The polymorphism information content (*PIC*) value ranged from 0.272 to 0.622. Potentially due to the substructure of the sampled population, three of the 14 microsatellite loci deviated from Hardy—Weinberg equilibrium (*HWE*). Further, loci C6, C22, and C31 could be amplified in *Cocobius fulvus* and *Encarsia japonica*, suggesting the transferability of these three polymorphic loci to other species of Hymenoptera.

## 1. Introduction

The diamondback moth (*Plutella xylostella*) is one of the most devastating pests for the global *Brassica* industry, and the estimated annual losses and management costs are approximately US $4–5 billion [1]. Traditional agrochemical-based management generates considerable drawbacks, such as pollution, habitat destruction, and loss of biodiversity. *P. xylostella* is attacked by natural enemies in all developmental stages; biological factors (e.g., parasitoids, arthropod predators, and pathogenic microorganisms, etc.) thus play a significant role in the integrated management of this species. There are more than 60 reported parasitoid species of *P. xylostella*, of which 12 have been widely applied for pest management in the field [2]. *Cotesia plutellae* is a larval parasitoid of *P. xylostella*, and its global distribution covers 38 countries on all continents except Antarctica [1]. As one of the most important parasitoids of *P. xylostella*, successful introduction of *C. plutellae* has been documented more than 20 times [1]. Parasitization of *P. xylostella* larvae by *C. plutellae* may increase their demand for food [3], extend the duration of the 4th-instar, and pupation (or the larva-to pupa metamorphosis) may be inhibited [4,5]. Before cocooning, *C. plutellae* moves out of the body of its host, eventually leading to its death.

Knowledge of the genetic variation and structure of the parasitoid populations favors parasitoid conservation and better pest control. Polymerase Chain Reaction (PCR) and molecular marker-based techniques are increasingly becoming recognized as valuable tools in ecological studies, and have been widely used in evaluating the genetic diversity of natural enemy populations [6]. Various advantages, such as high polymorphism, genome-wide distribution, co-dominant inheritance, and reproducibility, have made microsatellites one of the most widely applied types of marker in the field of molecular ecology [7]—especially in detecting genetic variation at the population level, evaluating population genetic diversity, and identifying genetic maps and kinship [8]. To date, however, no microsatellite-based studies have been performed on *C. plutellae*.

A comprehensive understanding of the biological characteristics, ecological characteristics, genetic variation, and interrelationships in different geographical populations of pests are prerequisite for the development of an effective pest control strategy [9]. Using molecular markers, we can understand the potential threat areas and effectively control the spread of pests [10]. So, the analysis and development of SSR markers with polymorphism has great application value and significance.

## 2. Material and Methods

### 2.1. Primer Design

A total of 19,309 microsatellite loci—among which 17,070 had complete motifs—were detected by MISA (MicroSAtellite identification) [11] based on the *C. plutellae* transcriptome dataset [12], downloaded from (National Center of Biotechnology Information) (NCBI) (http://www.ncbi.nlm.nih.gov/sra/DRX001445). Primer 3.0 [13] was used for primer design for the complete microsatellite loci, of which 85 trimers were randomly selected for validation with PCR.

### 2.2. Proved the Effectiveness of Primers

Primer effectiveness was verified on a lab-reared *C. plutellae* colony (Fuzhou, May 2014), and the subsequent identification of polymorphic loci and population genetic analysis was performed using individuals from wild *C. plutellae* populations (Fuzhou, April 2014). The *C. plutellae* were collected from nine 20 × 30 m plots with an average distance of 1.47 km (range, 0.65–3.68 km) between plots. Considering the arrhenotokous parthenogenesis of Hymenopteran species (i.e., diploid females develop from fertilized eggs and haploid males develop from unfertilized eggs) [14,15], only female *C. plutellae* individuals were used for population genetic analysis (*N* = 32).

Genomic DNA was individually isolated with the DNeasy Blood and Tissue Kit (QIAGEN, Hilden, Germany) following the manufacturer's instructions. We initially performed PCR to validate the effectiveness of the primer pairs. Polymorphism identification was based on the primer pairs and the expected product sizes. The forward primers of the validated primer pairs were linked with the universal primer *M-13* (TGT AAA ACG ACG GCC AGT) at their 5’ ends, and *M-13* labeled with FAM, HEX, or TAMRA fluorescent dye was also used in the genotyping system.

The total volume of each PCR mixture was 25 µL, containing 12.5 µL Mix (Promega), 0.2 µL forward primer, 0.8 µL reverse primer, and 0.8 µL *M-13*. The temperature conditions were at 94 °C for 10 min, and then 30 cycles at 94 °C for 30 s, Tm (the optimal annealing temperatures, see Table 1) for 45 s, 72 °C for 45 s, followed by eight cycles at 94 °C for 30 s, 53 °C for 45 s, 72 °C for 45 s, and a final extension at 72 °C for 10 min. 

### 2.3. Data Analysis

After testing by agarose gel electrophoresis (AGE), sizes of the amplification products were detected using an ABI 3730 sequencer (Applied Biosystems, Foster City, CA, USA). GeneMapper 4.1 (Applied Biosystems, Foster City, CA, USA) was used to assign alleles based on the sizes of PCR amplifications.

Fourteen polymorphic loci were isolated in this study. Number of alleles, expected heterozygosity (*H_O_*), observed heterozygosity (*H_E_*), and polymorphism information content (*PIC*) were calculated using genepop V4 (Montpellier, France) [16]. Hardy—Weinberg equilibrium (*HWE*) and linkage disequilibrium (*LD*) were also tested using genepop V4.

## 3. Results and Discussion

As one of the important indicators of population genetic variation, the number of alleles ranged from two to six for the 14 identified microsatellite loci in the tested population (Table 1). Comparable results can be found in recent studies. For example, the number of alleles per locus ranges from 2 to 19 for 30 female *Blastophaga javana* (Hymenoptera: Agaoninae) [17], the number of alleles ranges from 3 to 11 for *Trypoxylon (Trypargilum) albitarse* (Hymenoptera: Crabronidae) [18]; and the allelic number per locus varies from three to seven (*N* = 30) for *Pachycrepoideus vindemmiae* (Rondani) (Hymenoptera: Pteromalidae) [19]. A locus was considered to show low polymorphism with an associated *PIC* value of <0.25; and to show high polymorphism with an associated *PIC* value of >0.5. The polymorphism level of the 14 identified microsatellite loci in the present study were medium-to-high, with *PIC* values ranging from 0.272 to 0.622. The *H_E_* and *H_O_* values of the 14 microsatellite loci ranged from 0.123 to 0.316 and 0.141 to 0.281, respectively. Compared with the values of *H_E_*, the relatively lower *H_O_* values suggest a lack of heterozygosity in the examined population (Table 1).

## 4. Conclusions

Loci C6, C32, and C53 deviated significantly from *HWE*, and significant *LD* was observed between loci C6 and C32. Samples for this study were collected from different fields patchily distributed within the sampling site. Considering the weak migration capacity of *C. plutellae*, the structures of sub-populations from different fields might be responsible for such a deviation from *LD* and *HWE*.

**Figure 1:**
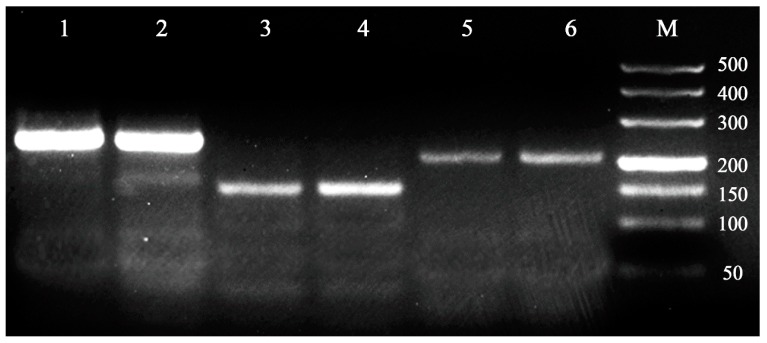
Gel electrophoretogram showing the effectiveness of three microsatellite loci (C6, C22, C31) in *C. fulvus* and *E. japonica*, with 1, 3, 5 for *C. fulvus*, and 2, 4, 6 for *E. japonica*. M: DL500 bp DNA marker.

The transferability of these primers (i.e., with detectable amplification products) was also verified in *Cocobius fulvus* and *Encarsia japonica*, and three of the fourteen microsatellite loci showed amplified products with the expected sizes (Figure 1). Therefore, the microsatellite loci characterized in this study will favor studies of genetic diversity and population structure of not only *C. plutellae*, but also other Hymenopteran parasitoids.

## Figures and Tables

**Table 1:** Characterization of the 14 polymorphic microsatellite loci developed for *C. plutellae.*

Locus	Repeat Motif	Primer Sequences (5’–3’)	*Tm* (°C)	*Na*	Size Range (bp)	*H_O_/H_E_*	Fis	Annotation
C6	CTG	F: AGAGCGGCAGTATCGTGAGT R: AGGAAAAGTCCTCAGCCTCC	53	5	245–260	0.250/0.316	0.2114 *	Putative uncharacterized protein
C19	AAT	F: CGCGAAAGAACGAATTTGAG R: TCACAGTATACGTCATTCCCAAG	54	5	129–141	0.141/0.129	−0.0941	Bifunctional protein FolD
C21	AAT	F: TCGCTAGAAAAAGTTTCGGC R: AATGAAGCAGGGTGAAATGC	53	4	232–241	0.172/0.129	0.1852	Putative uncharacterized protein
C22	CTG	F: CGCGACTCTCTGGCTCTACT R: TCAGGAGTCAGGAGTGGCTT	56	3	155–161	0.203/0.234	0.1352	cAMP responsive element-binding protein-like 2
C31	GAA	F: AAAACGTGACCAAAAGCTGG R: GGCCCGAGTACAAACAACTC	55	2	215–218	0.141/0.123	−0.1482	Putative uncharacterized protein
C32	CTG	F: TATGGGCGATAAAGGTGCTC R: AGGAAAAGTCCTCAGCCTCC	55	4	291–300	0.218/0.279	0.2202 *	Ceramide kinase
C51	TAT	F: AAAGGACGGGATAGATCGGT R: ACACTCAGGAATCCCACGAC	53	2	296–299	0.172/0.164	−0.0460	Putative uncharacterized protein
C53	TAT	F: GGCGAATTGGTTATGCTGAT R: TCGAAACATTGAGACAGCGT	53	6	210–257	0.188/0.276	0.3243 *	Putative uncharacterized protein
C54	TAT	F: TATCCTCTTCGCGCGTTATT R: AGGAACTCGTTTCCAACAGC	53	4	188–197	0.188/0.273	0.4572	Putative uncharacterized protein
C57	TCG	F: CCGGAACTGTTTTGTCACG R: CCGGAGTACGCTCTCAAGAC	52	4	124–133	0.250/0.266	0.0615	Putative uncharacterized protein
gi11	TTC	F: TTAATATAAACTGGCGGCGG R: CTCGGTCGACCAATGAAAAT	53	2	216–219	0.189/0.218	0.1429	Putative uncharacterized protein
gi16	TCT	F: TCCACTGCAAGCCATACAAG R: TGGTGATGTTGAGAAACCGA	53	2	126–129	0.203/0.248	0.1826	Putative uncharacterized protein
gi27	TAC	F: TGGATTTGCCACTACCATCA R: GTTGAAAGGGCCAATTTTGA	53	3	194–200	0.219/0.290	0.2485	Putative uncharacterized protein
gi29	TAG	F: TTAGTGGCGGCAGTGATAAT R: CCGTGTAACAAACCCCTGAT	53	3	244–250	0.281/0.298	0.0558	Putative uncharacterized protein

*Tm* annealing temperature of primer pairs; *Na* number of alleles; *Ho* observed heterozygosity; *H_E_* expected heterozygosity; * Significant deviation from Hardy—Weinberg equilibrium (*p* < 0.05).
